# The Polyamine Inhibitor Alpha-Difluoromethylornithine Modulates Hippocampus-Dependent Function after Single and Combined Injuries

**DOI:** 10.1371/journal.pone.0031094

**Published:** 2012-01-27

**Authors:** Susanna Rosi, Ryan Ferguson, Kelly Fishman, Antino Allen, Jacob Raber, John R. Fike

**Affiliations:** 1 Department of Neurological Surgery, University of California San Francisco, San Francisco, California, United States of America; 2 Department of Radiation Oncology, University of California San Francisco, San Francisco, California, United States of America; 3 Department of Physical Therapy and Rehabilitation Science, University of California San Francisco, San Francisco, California, United States of America; 4 Brain and Spinal Injury Center, University of California San Francisco, San Francisco, California, United States of America; 5 Department of Behavioral Neuroscience, Oregon Health and Science University, Portland, Oregon, United States of America; 6 Department of Neurology, Oregon Health and Science University, Portland, Oregon, United States of America; 7 Division of Neuroscience, Oregon National Primate Research Center (ONPRC), Oregon Health and Science University, Portland, Oregon, United States of America; National Cancer Institute, United States of America

## Abstract

Exposure to uncontrolled irradiation in a radiologic terrorism scenario, a natural disaster or a nuclear battlefield, will likely be concomitantly superimposed on other types of injury, such as trauma. In the central nervous system, radiation combined injury (RCI) involving irradiation and traumatic brain injury may have a multifaceted character. This may entail cellular and molecular changes that are associated with cognitive performance, including changes in neurogenesis and the expression of the plasticity-related immediate early gene Arc. Because traumatic stimuli initiate a characteristic early increase in polyamine metabolism, we hypothesized that treatment with the polyamine inhibitor alpha-difluoromethylornithine (DFMO) would reduce the adverse effects of single or combined injury on hippocampus structure and function. Hippocampal dependent cognitive impairments were quantified with the Morris water maze and showed that DFMO effectively reversed cognitive impairments after all injuries, particularly traumatic brain injury. Similar results were seen with respect to the expression of Arc protein, but not neurogenesis. Given that polyamines have been found to modulate inflammatory responses in the brain we also assessed the numbers of total and newly born activated microglia, and found reduced numbers of newly born cells. While the mechanisms responsible for the improvement in cognition after DFMO treatment are not yet clear, the present study provides new and compelling data regarding the potential use of DFMO as a potential countermeasure against the adverse effects of single or combined injury.

## Introduction

Uncontrolled exposure to radiation presents challenges unlike those encountered in a clinical situation, i.e. radiotherapy. The quality of radiation and dose homogeneity will likely be uncertain, and there will likely be a wide range of delivered doses and subsequent tissue/body effects. In addition, radiation effects might be complicated by other types of injury (trauma, burns, infection, etc.) that either occur at the time of irradiation or at some time thereafter. Given the growing worldwide threat of radiological/nuclear terrorism, a natural disaster or a nuclear battlefield, the concept of radiation combined injury (RCI) has been identified as a high priority research area [1]. While laboratory and some human data are available regarding whole body radiation exposure alone [2], there is a paucity of information regarding the magnitudes and mechanisms underlying the interactions between irradiation and other forms of injury, particularly in the central nervous system (CNS), or if the resultant damage can be prevented or ameliorated.

In the CNS, severe tissue injury generally occurs only after exposure to high radiation doses [3]. However, doses that do not induce significant tissue injury may predispose the brain to a higher vulnerability to a second insult, like trauma. One type of injury that can be induced by relatively low doses of irradiation, and that may be exacerbated in a RCI scenario, is cognitive injury or deficits in behavioral performance. In humans and animals, cognitive changes after irradiation alone or trauma alone often involve changes in hippocampus-dependent learning and spatial information processing [4]–[14]. While the mechanisms responsible for such changes are not well understood, they are likely multifactorial and may involve altered neurogenesis [8], [15]–[18] and the expression of the plasticity-related behaviorally-induced immediate early gene *Arc* (activity-regulated cytoskeleton-associated protein) [19], [20].

Naturally occurring polyamines (PAs: spermine, spermidine and putrescine) are essential polycations widely distributed in living organisms [21]. PAs have a variety of functions, including modulation of membrane receptor complexes and several intracellular signal transduction pathways [22]–[24]. Acute and transient increases in the PAs are a hallmark cellular response to various traumatic stimuli, and in the brain this may have a neuroprotective effect under certain circumstances. However, in the case of a persistent PA alterations such as those seen after some types of injury, like irradiation [25], [26], traumatic brain injury [27], [28], ischemia [29], [30], and lipopolysaccharide-induced inflammation [31], changes in brain PA response can be detrimental, leading to increased neuronal vulnerability [22].

In the brain, traumatic stimuli initiate a characteristic early increase in PA metabolism (i.e. the PA stress response) that is considered to be an integral component of a defensive cellular stress program [22], [23]. Increased PA catabolism with concomitant disruption of PA homeostasis has been shown to lead to a neurotoxic environment contributing to secondary injury after traumatic brain injury, and it was suggested that improved functional recovery may be associated with changes in PA metabolism [32]. The decarboxylation of ornithine to putrescine by the cytosolic enzyme ornithine decarboxylase (ODC) is the first and rate-limiting step in the polyamine biosynthetic pathway [33]. ODC is highly expressed in the hippocampal dentate gyrus [34] and is upregulated after traumatic brain injury [28]. Chronic oral administration of alpha-difluoromethylornithine (DFMO), an irreversible inhibitor of ODC, has been shown to reduce putrescine levels after a number of different treatment paradigms and to reduce specific morphologic and inflammatory changes in the brain that are associated with radiation injury [25], [26], [35], [36].

Currently there are no effective medical treatments to improve functional outcomes after traumatic brain injury [32]. Whereas an uncontrolled radiation exposure will result in a wide range of doses, we purposely, and arbitrarily, selected a relatively low whole body radiation dose that would not by itself cause radiation lethality. The present study represented a proof of concept study to determine if DFMO would modify the behavioral consequences of single insults or RCI. Considerable data are available showing that DFMO depletes PA levels [37]–[39], but for this study we focused exclusively on how DFMO affects factors associated with cognition.

## Results

**Figure 1:**
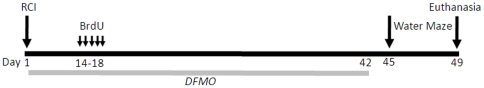
Experimental time line. Two-month-old C57BL/6 mice received whole body irradiation (4 Gy) and immediately after (∼15 min) received either controlled cortical contusion injury or sham craniotomy. Two weeks after injury animals were injected daily for 5 days with BrdU (100 mg/kg). DFMO was made available in the drinking water immediately upon recovery from surgery and for a total of 42 days and was stopped two days before cognitive testing in the Morris water maze. Animals were euthanized 30 minute after the last probe trial.

First, a DFMO toxicity study was performed. Animals that received vehicle (plain drinking water) only showed an average percentage weight gain of 18.6±4.7 (mean±SEM, n = 3) over the 8-week study period. Animals that received DFMO in drinking water showed average percentage weight changes of 14.6±5.3, 3.5±1.0, and −4.1±1.6 after 1%, 1.5% and 2% DFMO, respectively. There were no differences in blood cell profiles among the various groups (not shown). Animals that received either 1.5% or 2% DFMO experienced seizure-type activity when placed in the Morris water maze, while mice that received vehicle or 1% DFMO for 8 weeks showed no hyperactivity or seizures. Given the potential toxicity associated with an 8-week treatment with 1.5% or 2% DFMO, we chose the lowest DFMO dose (1%) for this study. Additionally we restricted total DFMO treatment time to 42 days, beginning immediately after injury and stopping two days prior to initiation of the cognitive testing (Fig. 1). After 42 days, and relative to sham treated mice that were treated with DFMO, the average percentage change in body weight across all injury groups was less than 5%; no animals in any group showed hyperactivity or seizure activity.

Radiation alone, trauma alone and RCI, with or without DFMO, were well tolerated by all animals. The controlled cortical impact model of traumatic brain injury is widely preferred because it generates many of the motor and cognitive impairments seen in TBI patients [40]. In the open head model, a portion of the skull is removed, and an impacting rod is driven into the dura to produce deformation of the cortex. Increasing the depth and velocity of the impact intensifies cortical cavitation as well as deficits in motor and behavioral function [41], [42]. The chosen impact depth (1.0 mm) used here was based on the previous studies performed in young adult mice where a 0.50 mm deformation produced mild injury, a 1.0 mm deformation produced moderate injury, and a 2.0 mm deformation produced severe injury [42].

In our study of moderate traumatic brain injury there was transient foreleg weakness in some animals; those effects subsided after 1-2 days. A cavitary cortical lesion was observed at the site of injury as previously described [13], and in most cases, the hippocampus proper was completely intact or showed only slight deformation. In all cases, the dentate gyrus was intact (data not shown).

Cognitive testing was performed 6 weeks after injury (radiation, trauma or RCI). With respect to swim speed during the visible platform sessions there was an irradiation x DFMO treatment interaction (*F* = 6.674, *p* = 0.002), with DFMO affecting swim speeds in sham but not in irradiated mice. While sham animals treated with DFMO swam slower than sham animals treated with vehicle (*F* = 11.715, *p* = 0.013; vehicle: 14.7 ± 0.4 cm/sec; DFMO: 12.4 ± 0.5 cm/sec), such differences were not observed when animals received irradiation (*F* = 0.067, *p* = 0.797). In contrast to irradiation alone, there were no effects of trauma alone, trauma alone + DFMO, RCI, or RCI + DFMO on swim speeds (data not shown).

Differences in swim speeds across groups could contribute to potential differences in time to locate the visible platform (latency), which is a measure of task learning. Therefore, mean swim speed during the visible session was included as a covariate in the analysis. During the visible sessions, there was an effect of DFMO treatment on latency to the target (*F* = 4.511, *p* = 0.039) and a radiation x DFMO treatment interaction (*F* = 7.155, *p* = 0.010). While sham mice treated with DFMO required less time to locate the visible platform location than vehicle treated sham mice (vehicle: 27.1 ± 1.2 sec; DFMO: 17.9 ± 1.5 sec), there was no difference in the time required to locate the visible platform between irradiated animals treated with DFMO and irradiated mice treated with vehicle (*F* = 0.067, *p* = 0.797). There were no effects of trauma alone, RCI, trauma + DFMO, or RCI + DFMO in terms of latency (data not shown).

For analysis of the hidden platform sessions, which measures spatial learning, we used the mean swim speed covariate also used during the visible platform sessions. There was a session x radiation x DFMO treatment interaction (*F* = 2.873, *p* = 0.015), mainly driven by an effect in sham-treated animals. When the sham-treated mice were analyzed separately, there was a session x DFMO treatment interaction (*F* = 2.293, *p* = 0.034), suggesting that at least in sham animals the effect of DFMO was not consistent across all testing sessions. The DFMO effect was most obvious in session 7, the first session on the second day, i.e. after the first probe trial (*F* = 8.885, *p* = 0.006). In that session, DFMO-treated sham mice required more time to locate the hidden platform location (vehicle: 12.1 ± 2.3 sec; DFMO: 23.0 ± 2.8 sec). This might be due to a DFMO-induced memory impairment. However, this could also be due to the effects from the first probe trial in which the animals might have learned there was no escape platform. There was also an effect of trauma on the mean latency across the hidden sessions (*F* = 6.364, *p* = 0.015), because injured mice required more time to locate the hidden platform location than sham animals (sham-injury: 15.1 ± 1.1 sec; trauma: 19.1 ± 1.0 sec). Together, these data showed that DFMO affected the swim speeds and the time required to locate the visible and hidden platform locations in sham but not in irradiated mice.

**Figure 2:**
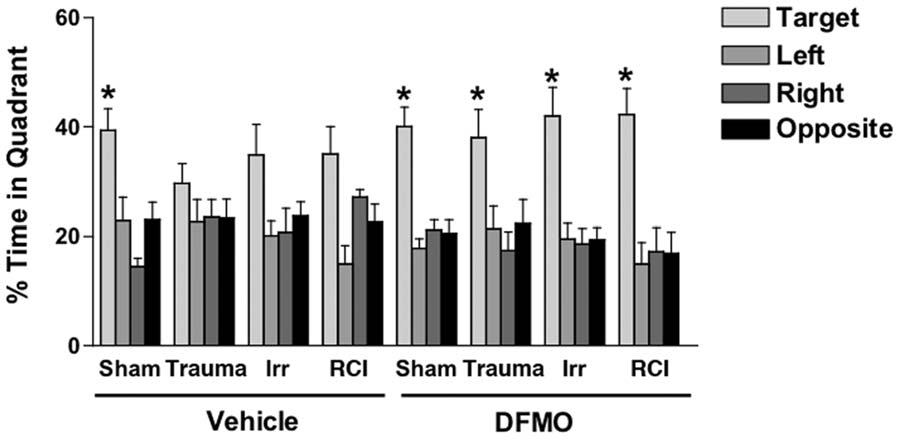
Spatial memory retention in the probe trial following the first day of hidden platform training. *p<0.05 or smaller versus any other quadrant. For details about the overall effect of quadrant and posthoc tests in individual groups, see text. Number of mice per experimental group: Control: n = 7; TBI: n = 8, Irradiation: n = 8, TBI/Irradiation: n = 8; DFMO control: n = 6; DFMO/TBI: n = 6; DFMO/Irradiation: 5; DFMO/TBI/Irradiation: n = 6.

**Figure 3:**
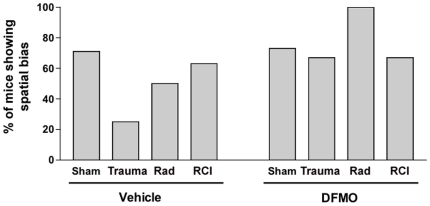
Percentage of mice that spent more time in the target quadrant than any other quadrant. For details, see text. Number of mice per experimental group: Control: n = 7; TBI: n = 8, Irradiation: n = 8, TBI/Irradiation: n = 8; DFMO control: n = 6; DFMO/TBI: n = 6; DFMO/Irradiation: 5; DFMO/TBI/Irradiation: n = 6.

Significant group differences were revealed during the probe trial (memory retention task) following the first day of hidden platform training (**Fig. 2**). Sham animals treated with vehicle showed spatial memory retention and spent more time in the target quadrant than any other quadrant (*F* = 9.336, *p* = 0.0003). In contrast, there was no preference for the target quadrant in any of the injury groups (**Fig. 2**). Similar patterns were seen in terms of the percentages of mice spending more time in the target quadrant than any other quadrant (**Fig. 3**), i.e. spatial bias. This latter analysis also showed that in the vehicle treated mice, the fraction of mice showing spatial bias after irradiation only and RCI was higher than that in the trauma only group.

Sham animals that received DFMO showed spatial memory retention in the first probe trial similar to what was seen in sham treated mice that were treated with vehicle (**Fig. 2**). That is, they spent more time in the target quadrant than any other quadrant (*F* = 15.124, *p*<0.0086). Mice that received trauma + DMFO (*F* = 4.367, *p* = 0.0161), irradiation + DMFO (*F* = 10.77, *p* = 0.0004) or RCI + DFMO (*F* = 9.904, *p* = 0.0006), all showed preference to the target quadrant, in contrast to what was seen in vehicle treated groups (**Fig. 2**). Relative to what was seen in vehicle treated animals, DFMO treatment increased the number of mice showing spatial bias in the trauma only and radiation only groups (**Fig. 3**).

During subsequent probe trials, there were no significant differences between sham-treated mice and mice that received trauma alone, irradiation alone, or RCI, whether or not they received DFMO (not shown). Thus, with sufficient additional training the spatial memory retention seen following just one day of hidden platform training could be overcome.

**Figure 4:**
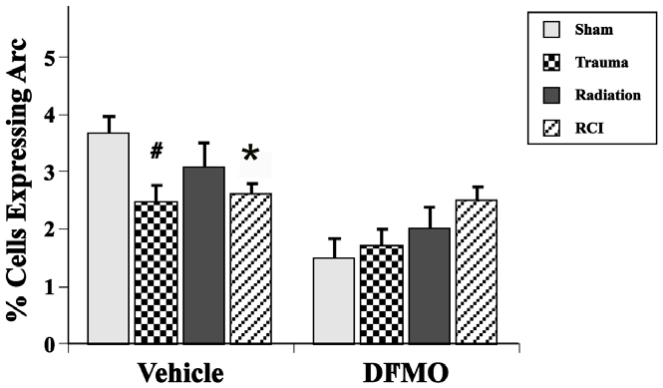
Percentages of hippocampal granule cell expressing Arc protein 30 min after the last probe trail of the Morris water maze. Two-way ANOVA revealed a significant interaction between drug treatment (DFMO vs vehicle) and injury (trauma, radiation, RCI). Animals treated with vehicle after trauma or RCI showed a significant reduction in the percentage of neurons expressing Arc relative to sham treated animals (#p<0.009, * p<0.02). There was no difference in the percentage of neurons expressing Arc between the different injury groups in the DFMO treated animals. Each treatment group consisted of 8–11 mice. Each bar represents a mean value and error bars are SEM.

Injury or DFMO treatment did not affect the average number of neurons in the dentate gyrus. In terms of the fractions of neurons expressing Arc protein, there was a significant interaction between drug treatment and injury (*F*_(1,57)_ = 4.17, *p*≤.01) (**Fig. 4**), with a significant effect of injury type in vehicle-treated **(**F_(3,25)_ = 3.12, p<0.04) but not in animals treated with DFMO. In vehicle-treated mice, post-hoc analyses showed significant reductions in the fraction of Arc+ neurons after trauma only (p<0.009) and RCI (p<0.02), but not after irradiation alone (**Fig. 4** **)**. In the contralateral hemisphere of animals that received injury plus vehicle, the numbers of neurons expressing Arc protein were slightly but not significantly reduced relative to sham mice (not shown). In the contralateral hemisphere of animals that received injury plus DFMO, the numbers of neurons expressing Arc protein were slightly but not significantly higher relative to sham mice (not shown).

**Figure 5:**
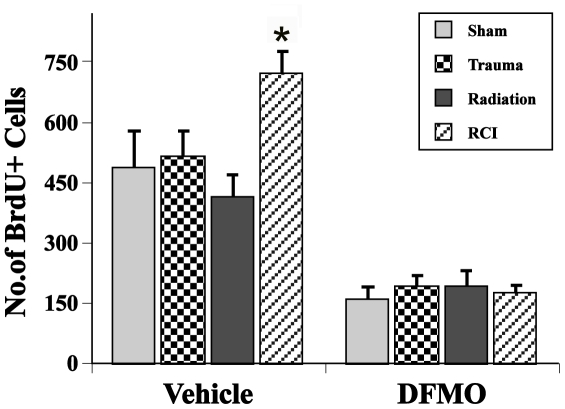
Total number of BrdU positive cells in the dentate subgranular zone. There was a significant interaction between drug treatment and injury. RCI animals treated with vehicle showed a significantly higher number of total BrdU compared to sham control animal (*p<0.05). In animals treated with DFMO there was no differences in the total number of BrdU positive cells among the different injury group. Each treatment group consisted of 8–11 mice. Each bar represents a mean value and error bars are SEM.

The presence of BrdU+ cells 4 weeks after BrdU injection represents the long-term survival of newly generated cells, independent of phenotype. Similar to what was seen for the Arc data described above, there was an interaction between drug treatment and injury in the ipsilateral hemisphere for BrdU+ cells (F(1,57) = 3.38, p≤.02), with a significant effect of injury type in vehicle-treated but not in animals treated with DFMO. (**Fig. 5**). There also was an effect of treatment alone (p<0.001) and of injury alone (p<0.001) on this endpoint (**Fig. 5**). In animals treated with vehicle, there was no difference in numbers of BrdU+ cells between sham-treated controls and mice that received trauma only or radiation only, with the numbers averaging 488±89, 517±59, and 417±50, respectively. However, after RCI the numbers of BrdU+ cells averaged 726±50 (p = 0.05, **Fig. 5**). In contrast to animals treated with vehicle, animals that received DFMO showed no differences in the total number of BrdU+ cells as a function of treatment, with all groups averaging about 150 BrdU+ cells. Because all DFMO groups had reduced BrdU counts relative to the corresponding vehicle groups, these data suggested that a drug dose of 1% had cytostatic effects independent of treatment. In the contralateral hemisphere we saw the same pattern as seen in the ipsilateral side (not shown).

**Figure 6:**
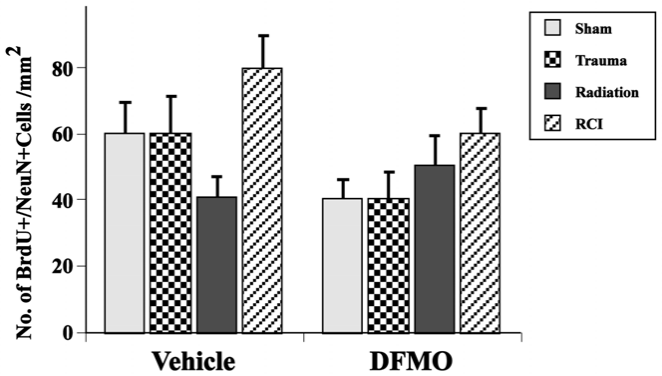
Total number of BrdU/NeuN positive neurons per mm^2^ in the dentate subgranular zone. Overall, there was no significant interaction between treatment and drug and no effect of the treatment alone or drug alone. In control mice treated with vehicle trauma alone induced no changes while radiation alone decreased the number of BrdU+/NeuN+ cells in the ipsilateral hemisphere. Animals from the RCI group showed an increase in the numbers of newly born neurons compared to radiation alone. Each treatment group consisted of 8–11 mice. Each bar represents a mean value and error bars are SEM.

In terms of newly born neurons (BrdU+/NeuN+) in the ipsilateral hemisphere, there were no significant effects of insult alone or drug alone (**Fig. 6**). With respect to newly born astrocytes (BrdU/GFAP+), there were effects of DFMO (p<0.001) and injury type (p<0.0001) on the numbers of cells in the dentate SGZ in the ipsilateral hemisphere (F**ig. 7**). DFMO reduced the number of newly born astrocytes as compared to the vehicle treatment. In addition, mice that received RCI had higher numbers of newly born astrocytes than sham or irradiated mice. In the contralateral hemisphere, such differences were not seen.

**Figure 7:**
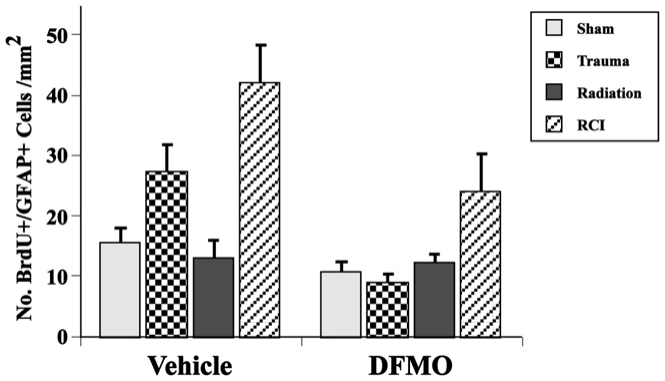
Total number of BrdU/GFAP positive cells per mm^2^ in the dentate subgranular zone. Overall, there was no interaction between drug (DFMO, vehicle) and treatment (trauma, radiation, RCI); however, independently both DFMO (p<0.001) and injury type (p<0.0001) had significant effects on the numbers of newly born astrocytes in the dentate SGZ. Each treatment group consisted of 8–11 mice. Each bar represents a mean value and error bars are SEM.

**Figure 8:**
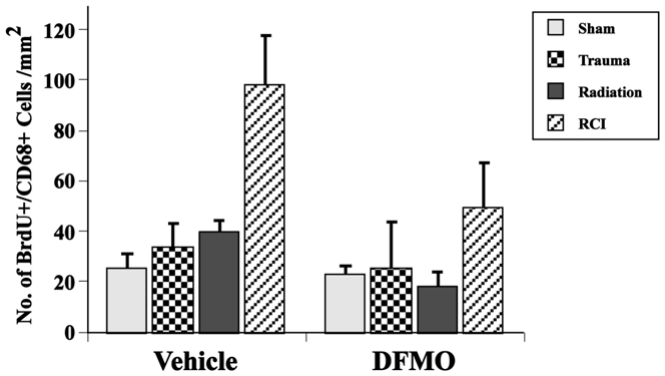
Total number of BrdU/CD68 (activated microglia) positive cells per mm^2^ in the dentate subgranular zone. Overall, there was no interaction between drug (DFMO, vehicle) and treatment (trauma, radiation, RCI); however, independently both DFMO (p<0.019) and injury type (p<0.0001) had significant effects on the numbers of newly born microglia. Each treatment group consisted of 8–11 mice. Each bar represents a mean value and error bars are SEM.

In mice that were treated with vehicle or DFMO there were no group differences seen in either hemisphere in terms of total number of activated microglia (CD68 only) (not shown). DFMO (p<0.019) and injury (p<0.0001) had an effect on newly born microglia (BrdU+/CD68+) in the ipsilateral hemisphere (**Fig. 8**). DFMO reduced the number of newly born microglia as compared to the vehicle treatment. In addition, mice that received RCI±DFMO had higher numbers of newly born microglia than sham, trauma, or irradiated mice.

## Discussion

The results of this study showed that: a) single and combined injuries induced variable degrees of hippocampus-dependent cognitive dysfunction and DFMO treatment ameliorated those effects; b) measurements of the plasticity-related immediately early gene Arc were consistent with the cognitive findings; and c) neurogenesis *per se* may not be a critical factor in the changes in cognitive performance with or without DFMO. Given there are virtually no data available addressing the concept of combined radiation and traumatic brain injury and how factors associated with long term behavioral performance are affected in such a scenario, the present study provides new and compelling data regarding the potential use of DFMO in this context. We recognize that a potential countermeasure against RCI will probably be administered some time post exposure, after adequate triage mechanisms have been implemented. However, laboratory studies such as this one need to be performed first to determine if a given agent has any promise at all against single or RCI.

Treatment with 1% DFMO for a total of 42 days did not induce any adverse effects in terms of body weight or blood cell profiles, suggesting the compound was minimally toxic. However, the finding that sham animals treated with DFMO swam slower than animals treated with vehicle indicates there were some physical decrements induced by prolonged treatment with the drug. The fact that irradiation resolved or prevented this effect is interesting but not yet understood. The same type of paradoxical effect was seen in the hidden platform sessions. This suggested that DFMO might induce a learning impairment that was reversed after irradiation. The significance of these effects and how they develop is not known, but their magnitude does not indicate a significant toxicity associated with chronic DFMO treatment. Other investigators have previously addressed potential adverse effects of chronic DFMO treatment on cognition [43], [44], and it was concluded that acute or chronic DFMO treatments might have very different behavioral effects [44]. Chronic DFMO treatment for 54 days was reported to affect spatial learning and memory in rats, but that study used a much higher concentration of DFMO in the drinking water (3%) than used in the present study.

Hippocampus dependent cognitive impairments were quantified using the Morris water maze [45] and a paradigm involving a probe trial at the end of each day of multiple hidden platform training sessions [46]. In animals that received vehicle, only sham-treated mice showed significant preference for the target quadrant in the first probe trial, and animals that received trauma only were most affected (**Fig. 2**). Analysis of spatial bias, which quantifies the proportion of mice in a given group that exhibit spatial memory retention, clearly showed mice that received trauma alone were the most seriously affected (**Fig. 3**). Those changes were not seen in subsequent probe trials suggesting that even cognitively impaired animals could successfully perform the water maze task with additional training. This latter finding highlights the strength of using multiple probe trials in the design of the water maze task, a result previously reported in the assessment of RCI [20] and after irradiation of mice expressing different human apoE isoforms [47] or mice lacking the gene for extracellular superoxide dismutase [46].

The present data showed that DFMO had a significant positive effect in terms of the retention of spatial memory. Regardless of injury type, all animals that received DFMO showed significant preference to the target quadrant, (**Fig. 2**). Analysis of spatial bias (**Fig. 3**) confirmed the observation that DFMO effectively reversed cognitive impairment after all injuries. Changes in spatial bias between vehicle-treated and DFMO-treated mice were particularly obvious after trauma alone. These findings, while representing only one DFMO dose and treatment schedule, suggest that further studies of this agent are warranted to determine its usefulness as a potential countermeasure for single and combined injuries.

There are a variety of factors that may impact or influence the development or severity of cognitive impairment seen after single or combined injuries. Three of these, Arc expression, neurogenesis and neuroinflammation, have been used previously to assess how irradiation affects factors associated with cognitive performance with or without traumatic brain injury [19], [20], [48], [49]. Gene expression associated with learning and memory produces *de novo* synthesis of proteins, like Arc, which alter synaptic strength and are essential for memory processes [50]–[53]. Arc expression plays an important role in the neuroplastic mechanisms critical to memory consolidation [54], and inhibition of Arc protein expression impairs the consolidation of long-term memory during Morris water maze testing [55]. Therefore, the significant reduction of Arc expression seen here in vehicle-treated animals that experienced trauma alone and RCI (**Fig. 4**), may partially explain the inability of the animals to properly recall the location of the platform during the first probe trial (**Fig. 2**).

However, the absolute number of neurons expressing Arc is not a simple predictor of cognitive performance. Sham treated mice that received DFMO had significantly fewer Arc+ neurons when compared to sham treated mice that received vehicle, yet the cognitive performances of those groups were comparable (**Fig. 2**). Whether the reduction in the number of neurons expressing Arc in DFMO treated animals represents a simple ‘resetting’ of the system to a lower baseline level or indicates that Arc expression is not an adequate predictor of cognitive performance is not known. However, the markedly different pattern of Arc expression in the various DFMO treatment groups (trauma, irradiation, RCI) relative to that seen in groups receiving vehicle, suggests that it is the former rather than the latter. That is, after injury plus DFMO, where the average fractions of Arc+ cells were higher than their sham-controls, there was an overall improvement in cognitive performance compared to injury plus vehicle, where cognitive performances were impaired and the average fractions of Arc+ cells injury groups were lower than their sham-control.

Another factor that has been associated with cognitive performance is hippocampal neurogenesis, and data exist showing that neurogenic cells in the hippocampus are very radiosensitive, and are reduced by doses that are below the threshold for the lethal hematopoeitic or gastrointestinal radiation syndromes [8], [15]–[18]. Furthermore, changes in neurogenesis are associated with hippocampal dependent behavioral deficits [5]–[10], [20], [56]–[58]. Because traumatic brain injury shares specific neuropathologic characteristics with ionizing irradiation (i.e. reduced neurogenesis, reduced Arc expression, inflammation, hippocampal-dependent cognitive impairment) [11]–[14], [20], the impact of the separate stimuli may work in concert to cause more injury than would be expected by a simple summation of effects. The relationship between neurogenesis and cognitive function is complicated and very well could be context-dependent, that is, it may differ depending upon the experimental conditions being investigated [20].

With respect to the numbers of newly generated cells, independent of phenotype (i.e. BrdU+ only), only mice that were treated with vehicle and received RCI showed any significant change relative to controls or other treatments (**Fig. 5**). Animals that received irradiation only had fewer BrdU+ cells but that change was not significant. DFMO significantly reduced BrdU labeling in all treatment groups to about the same level, a finding consistent with the known cytostatic effects of the compound [59]. In terms of newly born neurons (BrdU+/NeuN+) there were no significant differences across treatments in mice that received either vehicle or DFMO. The trend toward a decrease after irradiation plus vehicle and an increase in RCI plus vehicle, appeared to be change after DFMO treatment, but the magnitudes and lack of significance precludes any definitive conclusions. Given that, the data suggest that the generation of newly born neurons *per se* may not be a critical factor in the changes in cognitive performance observed here, a finding in support of a previous report on RCI [20].

There were generally fewer newly born astrocytes in the dentate SGZ in animals treated with DFMO than in mice treated with vehicle, and in both treatment groups the numbers of cells were significantly affected by injury type (**Fig. 7**). It will require further study to understand the implications associated with these changes, and if they are relevant to behavioral performance.

Given that polyamines have been found to modulate inflammatory responses in the brain [31], and inflammation has been shown to affect neurogenesis [15], [60]–[63] and Arc expression after irradiation [19], we hypothesized that DFMO may affect cognitive performance after single or combined treatment via changes in neuroinflammation. We monitored inflammatory changes by quantifying numbers of microglia, a cell type commonly assessed as a measure of neuroinflammation after radiation exposure [15], [60]–[63]. While there were no differences across treatment and injury groups with respect to total numbers of activated microglia, there were generally fewer newly born (BrdU+) activated microglia after DFMO treatment when compared with animals treated with vehicle (**Fig. 8**). While these results are compatible with a generalized reduction of inflammation in DFMO-treated mice, the data are not sufficient to made definitive conclusions. Additional studies, and in particular the measurement of more factors associated with inflammation, are required to determine if the overall effects of DFMO on cognitive performance are mediated by inflammatory processes.

The mechanisms responsible for the improvement in cognition after DFMO treatment are not yet clear, and future studies will be required to determine if those effects are mediated by, or independent of, actual polyamine levels in the hippocampus. Nonetheless, the fact that such a reversal is observed, particularly after trauma, is noteworthy and potentially very important. Further studies with DFMO are needed to determine not only its potential usefulness in managing traumatic brain injury alone but also its potential as a countermeasure against RCI.

## Methods

### Ethics statement

Mice were housed and cared for in compliance with the United States Department of Health and Human Services Guide for the Care and Use of Laboratory Animals and institutional IACUCs.

Seventy-two male C57BL/6 mice (Jackson Laboratory, Bar Harbor, ME), 2 months old, were used. Twelve mice were used for a pilot DFMO dosing study and the rest of the animals in each injury paradigm were randomly distributed into 2 cohorts, that received either regular drinking water (vehicle) or water with 1% DFMO. Within each cohort there were 4 treatment categories: 0 Gy/sham surgery (Sham, n = 19), 4.0 Gy/sham surgery (Radiation, n = 13); 0 Gy/trauma (Trauma, n = 14); 4.0 Gy/trauma (RCI, n = 14). Animals were group housed (4 mice/cage) throughout the study. Mice were maintained on a 12-h light-dark cycle, and irradiations, induction of trauma and behavioral testing were done during the light period. All procedures (sham surgery, trauma and irradiation) were performed between 10 am and 2 pm, and all behavioral training and testing were done on all animals at the same time of day. All animals were provided food and water ad libitum.

### Irradiation and Traumatic Brain Injury

Whole body irradiation of unanesthetized mice was done using a ^137^Cs irradiator (Gamma Cell 3000, MDS Nordion Inc., Ottawa Ontario, Canada). Animals were irradiated individually in a specially designed restrainer that fit into the irradiator and allowed animals to minimally move around. Dosimetry was performed using film exposure within the cesium irradiator under the same geometry used for the animal treatments. The film readings were calibrated against a range of doses obtained using a linear accelerator. The dose chosen for this study was 4.0 Gy, a dose that does not induce significant changes in the gut or bone marrow in mice. The total time of irradiation was approximately 1 minute.

Immediately after irradiation, mice were anesthetized with 4% isoflurane. Anesthesia was maintained via a non-rebreathing apparatus connected to a nose cone on the stereotaxic head frame (Kopf, Tujunga, CA). Ointment was applied to the eyes to protect vision, and heads were shaved with an electric clipper. The skin was prepped with betadine ointment, and a midline incision was made through the scalp. A circular craniotomy, 3.5 mm in diameter was made in the left parietal skull between the bregma and lambda, 0.5 mm lateral to the midline. The skullcap was carefully removed without disruption of the dura. All mice, regardless of injury type, were subjected to this surgical procedure. Mice that were randomly selected for the trauma only (no irradiation) or RCI treatment groups were subjected to a controlled cortical impact ([13], [20]. The lesion was produced with a pneumatic impact device using a 3-mm-diameter convex tip, mounted 20^0^ from the vertical to account for the curvature of the skull. The contact velocity was set at 4.5 m/s with a deformation 1.5 mm below the dura, producing a moderately severe lesion to the cortex without encroaching on the hippocampus. After the procedure, the scalp was sutured and each animal received a subcutaneous injection of warm physiologic saline (1 ml) to prevent dehydration. During surgery and subsequent recovery, body temperature was maintained with a circulating water heating pad.

### DFMO Treatment

DFMO was generously provided by Dr. Victor A. Levin. DFMO is stable in drinking water for prolonged period of time (months) providing there is no growth or contamination by microorganisms (V. Levin, personal communication). A large number of studies are available showing that DFMO can be given in this manner, and is effective at concentrations ranging from 0.5–3% [31], [38], [64]–[66]. It has been reported that such chronic treatment with DFMO can induced strain-dependent hyperactive behavior and seizure activity upon additional stimulation (e.g. noise) [67]. Thus, we performed an initial dose ranging study in small groups of uninjured mice to determine the dose to be used in our subsequent RCI study. Groups of three mice received either vehicle, or 1%, 1.5% or 2% (w/v) DFMO dissolved in drinking water. The DFMO solutions were changed weekly. To assess general health of animals, mice were weighed 2×/week for 8 weeks, and water maze training and testing was initiated to determine if DFMO affected behavioral performance. Blood was obtained at 8 weeks when the animals were euthanized.

Once the DFMO treatment dose was established (i.e. 1%), single or combined treatments were initiated either with or without drug. The DFMO was administered as described above. DFMO was made available to the mice immediately upon their recovery from surgery and for a total of 42 days (**Fig. 1**). DFMO treatment was stopped during the 5 days of cognitive testing.

### BrdU Injection

Fourteen days following sham injury or traumatic brain injury, all mice received daily injections of BrdU (100 mg/kg) for 5 consecutive days. Four weeks after the first BrdU injection mice underwent Morris water maze training and testing and then they were euthanized and tissues were collected for analysis of neurogenesis and Arc protein expression (**Fig. 1**).

### Morris Water Maze

Assessment of hippocampus-dependent cognitive performance was started 6 weeks after irradiation, trauma or RCI using the Morris water maze test as previously described [20], [68]. Briefly, a circular pool (diameter 140 cm) was filled with opaque water (24°C) and mice were trained to locate a platform (luminescence: 200 lux). To determine if treatment affected the ability to swim or learn the water maze task, mice were first trained to locate a clearly marked platform (visible platform, Days 1 and 2). Mice were subsequently trained to locate the platform when it was hidden beneath the surface of the opaque water (Days 3–5). Hidden platform training (acquisition) required the mice to learn the location of the hidden platform based on extra-maze cues. For both visible and hidden platform paradigms, there were 2 daily sessions which were 2 hr apart. Each session consisted of 3 trials (with 10-min inter-trial intervals). A trial ended when the mice located the platform. Mice that failed to locate the platform within 60 sec were lead to the platform by placing a finger in front of their swim path. Mice were taken out of the pool after they were physically on the platform for a minimum of 3 sec. During visible platform training, the platform was moved to a different quadrant of the pool for each session. For the hidden platform training, the platform location was kept constant. Mice were placed into the water facing the edge of the pool in one of 9 randomized locations. The start location was changed for each trial. Swimming patterns were recorded with the Noldus Ethovision video tracking system (Ethovision XT, Noldus Information Technology, Wageningen, Netherlands) set at 6 samples/second. The time to locate the platform (latency) was used as a measure of performance for the visible and hidden sessions.

To measure spatial memory retention, probe trials (platform removed) were conduced 1 hr after the last trial on each day of hidden platform training (i.e. 3 separate probe trials). For the probe trials, mice were placed into the water in the quadrant opposite from the target quadrant. The time spent in the target quadrant, i.e. where the platform was previously located during hidden platform training, was compared to the time spent in the 3 non-target quadrants. In addition, the percentage of mice from each experimental cohort that spent more time in the target quadrant than any other quadrant was calculated.

After the last probe trial, mice were returned to their home cages. Thirty min later, mice were killed by cervical dislocation and decapitated. The 30 min interval was chosen because at that time, behaviorally induced Arc protein can be detected within the DG [19], [20]. Brains were removed quickly (within 60 sec) and frozen in −70°C isopentane.

### Histological Procedures and Analysis

The brains from multiple animals were blocked together and cryosectioned [19], [20] such that each slide contained sections from one animal from each of the experimental conditions: Sham, Radiation, Trauma, and RCI. All slides were stored at −70°C until processed for immunocytochemical analysis. Brain sections were taken from the medial portion of the dorsal hippocampus (anteroposterior ∼2.92–4.0 mm from bregma). Quantitative assessment of Arc protein was performed using methods previously reported by us in detail [19], [20], [52]. Tissue sections were fixed in 2% paraformaldehyde, and after blocking with a tyramide signal amplification kit (TSA; PerkinElmer Life Science, Emeryville, CA), they were incubated in polyclonal rabbit anti-Arc antibody for 48 h at 4°C, (1∶500; rabbit anti-Arc a gift from Paul F. Worley, Johns Hopkins University School of Medicine, Baltimore, MD). Sections were then incubated with an anti-rabbit biotinylated secondary antibody (Vector Laboratories) for 2 h at room temperature, followed by amplification with an avidin-biotin system for 45 minutes. Staining was reveled using a cyanine-3 (CY3) TSA fluorescence system (PerkinElmer).

Microscopic imaging for Arc protein was performed at 20× magnification using a Zeiss AXIO IMAGER Z1 microscope with motorized Z-drive for transmitted light and epi-fluorescence [19]. For each endpoint, two coronal sections per mouse were randomly selected from the dorsal hippocampus (1.7 to 2.3 mm posterior from Bregma) and used to reconstruct mosaics of the ipsilateral and contralateral DG. Each mosaic was formed from 6–8 Z-stack (1.0 µm optical thickness/plane) images.

To determine if irradiation, traumatic brain injury and/or combined injury resulted in variations in granule cell density, the numbers of cells per mm^2^ were counted from selected regions of the ipsilateral DG in one representative section from each animal in the study.

Arc-positive neurons had perinuclear-cytoplasmic staining surrounding the cell and visible in at least three plains together with the cell nucleus across the z-stack [19], [20], [52]. To avoid classification errors, we carefully verified that the staining belonged to the cell of interest by checking the nuclear counterstaining. The numbers of Arc positive neurons/mm^2^ characterized by these criteria were determined in the enclosed and free blades of the DG [19], [20], [52]. We then calculated the percentage of neurons expressing Arc protein for each animal [19], [52]. The only studies involving Arc and cognitive performance after irradiation and traumatic brain injury have been restricted to the DG [19], [20]. Given that the kinetics of Arc expression in the DG is different from the other hippocampal subfields [51], and because they have not yet been characterized in mice, in the current study we restricted our analysis to the DG.

To determine the effects of single or combined treatment on the survival of newly born cells in the dentate subgranular zone (SGZ), sections were prepared as above and immunostained as previously described [15], [16]. Newly born cells were stained using rat anti-BrdU (1∶10; Oxford Biotechnology, Kidlington, Oxford, UK), neuronal cells were stained with biotinylated mouse anti-NeuN (1∶200; Chemicon, Temecula, CA, USA), astrocytes with anti-glial fibrillary acidic protein (GFAP, Pharmigen, San Diego, CA, USA), and activated microglia with anti-CD68 (Sertotec Inc., Raleigh, NC); cell specific staining was revealed respectively with Texas red (Vector, Burlingame, CA) and Alexa Fluor 499 or 594 (Molecular Probes, Eugene, OR, USA).

To calculate the numbers of BrdU-positive (BrdU+) cells in the SGZ, 12 sections of a one-in-six series were scored per animal [16]. All counts were limited to the dentate granule cell layer and a 50 µm border along the hilar margin that included the SGZ; cell counts were obtained for both hemispheres. BrdU+ cells displaying the various lineage-specific phenotypes were counted in two representative sections and the values converted to numbers of cells/mm^2^.

### Statistics

Water maze performance was analyzed using SPSS software using repeated measures ANOVAs to compare visible and hidden water maze learning curves (radiation, trauma, RCI, and treatment as between subjects factors and session as within subjects factor). Tukey-Kramer *post hoc* tests were used when appropriate. For analysis of performance in the water maze probe trials, one-way ANOVAs were used along with Dunnett's posthoc tests comparing each non-target quadrant to the target quadrant. Statistical analyses for the immunohistochemistry cell counts were performed using GraphPad Prism software (La Jolla, CA 92037 USA). Two-way ANOVA was used to test the main effects of treatment (DFMO or vehicle) and injury (radiation, trauma or RCI and potential interaction between treatment and injury). When the interaction between injury and treatment was significant (p<0.05), a step-down procedure for multiple comparisons would follow with two separate one-way ANOVAs. Different injuries or treatments were used as the independent variables and the percentages of cells from each category described above as the dependent variables. When the overall ANOVA was significant (p<0.05), individual between-groups comparisons were performed with Bonferroni post hoc to correct for multiple comparisons. Data for the ipsilateral and contralateral hemispheres were analyzed separately [20].
